# Development of the Stitching—Oblique Incidence Interferometry Measurement Method for the Surface Flatness of Large-Scale and Elongated Ceramic Parts

**DOI:** 10.3390/s25175270

**Published:** 2025-08-24

**Authors:** Shuai Wang, Zepei Zheng, Wule Zhu, Bosong Duan, Zhi-Zheng Ju, Bingfeng Ju

**Affiliations:** The State Key Laboratory of Fluid Power and Mechatronic Systems, Zhejiang University, Hangzhou 310058, China; 11825037@zju.edu.cn (S.W.); 22325067@zju.edu.cn (Z.Z.); wulezhu@zju.edu.cn (W.Z.); zhizheng.22@intl.zju.edu.cn (Z.-Z.J.); mbfju@zju.edu.cn (B.J.)

**Keywords:** oblique-incidence interferometry, sub-aperture stitching, surface flatness, large-scale ceramic guideway, precision measurement

## Abstract

With the increasing demand for high-performance ceramic guideways in precision industries, accurate flatness measurement of large-scale, rough ceramic surfaces remains challenging. This paper proposes a novel method combining oblique-incidence laser interferometry and sub-aperture stitching to overcome limitations of conventional techniques. The oblique-incidence approach enhances interference signal strength on low-reflectivity surfaces, while stitching integrates high-resolution sub-aperture measurements for full-surface characterization. Numerical simulations validated the method’s feasibility, showing consistent reconstruction of surfaces with flatness values of 1–20 μm. Experimental validation on a 1050 mm × 130 mm SiC guideway achieved a full-surface measurement with PV 2.76 μm and RMS 0.59 μm, demonstrating high agreement with traditional methods in polished regions. The technique enabled quick monitoring of a 39-h lapping process, converging flatness from 13.97 μm to 2.76 μm, proving its efficacy for in-process feedback in ultra-precision manufacturing.

## 1. Introduction

With the rapid development of modern industry, the performance requirements for guideways in mechanical systems have become increasingly demanding. Traditional metal guideways are progressively unable to meet the needs for high precision, heavy loads, and operation under special environmental conditions. In response to these challenges, ceramic materials have gained attention as viable candidates for high-performance guideway applications. Ceramics such as alumina (Al_2_O_3_) and silicon nitride (Si_3_N_4_) offer desirable properties including high hardness, wear resistance, thermal stability, corrosion resistance, and rigidity, making them suitable for use in fields such as mechanical engineering, semiconductor manufacturing, and medical devices [1,2,3]. In semiconductor manufacturing in particular, ceramic guideways have become indispensable components in critical equipment such as lithography systems, due to their advantageous physical and chemical characteristics. For large-scale, elongated structural ceramic components analogous to guideways, performance depends not only on the intrinsic material properties but also on the machining accuracy [4]. Among the relevant parameters, flatness is a critical indicator of machining quality for ultra-precision ceramic components. It directly affects the motion accuracy and stability of planar motion pairs and is essential to the high-precision performance of mechanical equipment. Therefore, controlling flatness with high accuracy is crucial during the fabrication of such components.

Flatness refers to the deviation of the macroscopic height variation of a substrate surface from an ideal plane. It quantifies the amount by which an actual surface departs from perfect flatness and is used to describe the degree of surface uniformity. Common instruments for flatness measurement include the knife-edge [5,6], coordinate measuring machine (CMM) [7], laser interferometer [8], phase-shifting laser interferometer [9], electronic level [10,11], autocollimator [12,13], and step gauge [14]. The selection of a specific method depends on the size and required accuracy of the surface under inspection, as different techniques are suited to different measurement scenarios. The knife-edge method, with a measurement accuracy on the order of micrometers, is suitable for preliminary assessment of small surfaces but offers low sampling density. Coordinate measuring machines (CMMs) provide sub-micrometer accuracy and wide measurement ranges, making them suitable for high-precision flatness evaluation of various parts, with sampling density adjustable as needed. Laser interferometers also offer micrometer-level accuracy and are suitable for large-area surfaces, with flexible sampling intervals. Electronic levels typically offer an accuracy ranging from tens of micrometers to several millimeters per meter. Although applicable to surfaces of various sizes, multiple measurements are often required for large surfaces, resulting in relatively low sampling density. Autocollimators achieve micrometer-level accuracy and are appropriate for medium to large surfaces, with moderate sampling density. Step gauges, also with micrometer-level accuracy, are suitable for small to medium-sized surfaces, with sampling density determined by the length of the bridge plate. Compared to the instruments listed above, phase-shifting laser interferometers offer superior performance in both accuracy and sampling resolution. They can achieve root-mean-square (RMS) repeatability at the nanometer level and sampling intervals as fine as 0.1 mm. Depending on the aperture of the interferometer, they are suitable for measuring surfaces of various sizes and can acquire data from millions of measurement points simultaneously. However, due to their high sensitivity to surface roughness, such instruments are generally applicable only to polished surfaces.

The measurement of large-scale, elongated surfaces presents significant challenges. First, for ultra-precision flat guideway components, the flatness tolerance often needs to reach the sub-micrometer or micrometer level, rendering low-precision, small-range methods such as gap-based techniques unsuitable. Second, these ultra-precision ceramic components require accurate surface profile correction during grinding and polishing. As a result, measurement methods with low sampling density, limited repeatability, and high operational complexity—such as laser interferometry, autocollimation, electronic leveling, and step gauges—are inadequate for providing effective guidance in surface correction processes. Furthermore, for ultra-precision ceramic components with sizes exceeding 1 m, the measurement accuracy of methods such as CMMs, laser interferometers, autocollimators, and electronic levels tends to degrade with increasing measurement range. Additionally, the required measurement time increases proportionally with sampling density, making high-resolution measurements time-consuming. Moreover, during the grinding stage, due to relatively poor surface roughness, ceramic materials such as Al_2_O_3_ and SiC are generally unsuitable for measurement using phase-shifting laser interferometry.

To address these challenges, many researchers have proposed various approaches. Lu et al. [15] segmented long surfaces into overlapping short sections for separate measurement and stitching. Using a custom-built system with a laser displacement sensor and a reference plate, they measured the straightness profile of a 500 mm-long machine tool guideway with a flatness deviation of approximately 92 μm. The maximum variation among three measurements was about 4.47% of the average dimensional tolerance. However, this method is limited to straightness measurement and does not provide comprehensive surface profile information. Li et al. [16] developed a flatness measurement machine based on a high-precision reference mirror and an error separation system. The system achieved a repeatability better than 0.1 μm and was used to reduce the flatness deviation of a 700 mm-long metal guideway surface from PV 2.617 μm to PV 0.825 μm. Zhou et al. [17] applied a stitching-based measurement approach using a high-precision flat mirror and displacement sensors, combined with a coordinate transformation algorithm. They measured the flatness of a 1400 mm × 500 mm metal optical surface and guided its error convergence from PV 7.54 μm to PV 2.98 μm. However, both methods rely on long glass reference surfaces with sub-micrometer or tens-of-nanometers flatness, which are difficult to manufacture and measure [18]. Li et al. [19] also proposed a laser interferometry-based method that utilizes the angular stability between adjacent guideway segments for long-range straightness stitching. The deviation from direct measurement was less than 10 μm over a 30 m length, and about 10 μm compared with an electronic level when applied to a 72 m guideway. This method is suitable for very long guideways, but its accuracy is limited. Rui et al. [20] proposed a grazing-incidence stitching interferometry method with an astigmatism-based self-calibration algorithm for measuring rotationally symmetric circular polishing pads. While their approach achieved high precision (0.17 μm flatness error) in stable workshop environments, it exclusively addressed axisymmetric surfaces and relied on rotational scanning, thus lacking applicability to high-aspect-ratio elongated components. Furthermore, it concentrated on single measurements and did not experimentally demonstrate rapid detection during surface convergence processes. Lai et al. [18] designed and manufactured a K9 glass guideway for use in a precision profilometer. The guideway was fabricated using computer-controlled optical machining and magnetorheological polishing. Flatness measurements of surfaces with dimensions 420 mm × 80 mm and 420 mm × 170 mm were conducted using a 24-inch Zygo Verifire MST interferometer (Zygo Corporation, Middle field, Middlefield, CT, USA), and the reference surface achieved a PV flatness of 98 nm. However, this approach is only applicable to optical surfaces and does not address the measurement of non-optical ceramic surfaces. Despite their effectiveness in specific applications, these techniques are not suitable for achieving high-precision and repeatable measurements on unpolished elongated ceramic surfaces.

To address the challenge of rapid surface flatness measurement for low-reflectivity, large-scale and high-aspect-ratio ceramic parts, this paper presents a measurement method that combines oblique-incidence laser interferometry with sub-aperture stitching. The oblique-incidence laser interferometry technique enhances the interference signal strength on low-reflectivity ceramic surfaces, thereby improving measurement accuracy. The sub-aperture stitching technique integrates high-precision measurements from multiple sub-apertures to enable a comprehensive and accurate assessment of large surfaces. This combined approach overcomes the limitations of conventional measurement techniques, such as insufficient accuracy, low sampling density, and the inability of standard laser interferometers to adapt to the full ultra-precision machining process. It provides a novel and effective solution for high-precision flatness measurement of large-scale, elongated components, such as ultra-precision ceramic guideways.

The structure of this paper is organized as follows: Section 2 provides a detailed explanation of the fundamental principles of the proposed measurement method. In Section 3, a mathematical model is established and numerical simulations are conducted to verify the feasibility and effectiveness of the method. Section 4 present the design and implementation of a series of measurement experiments, in which the flatness of a silicon carbide (SiC) ceramic guideway with a length of 1050 mm and a width of 130 mm is evaluated to assess the performance of the proposed method. The results are compared with those obtained using conventional measurement techniques. Finally, Section 5 summarizes the research findings and discusses the advantages and application prospects of the proposed measurement approach.

## 2. Method

**Figure 1:**
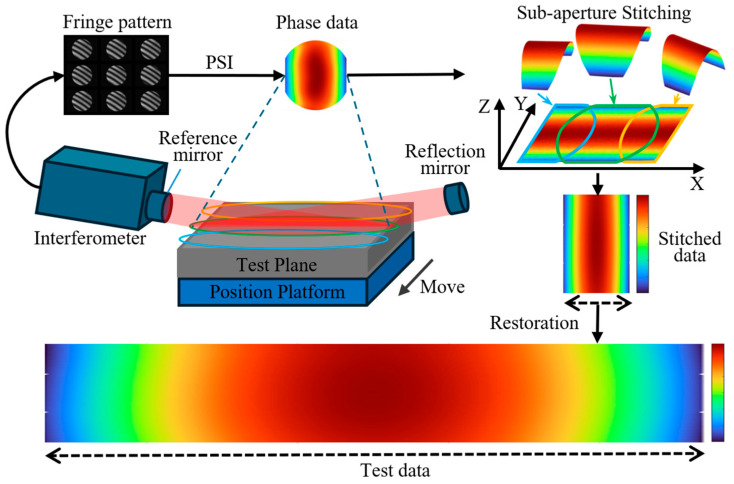
Principle of Splicing-Based Oblique-Incidence Laser Interferometric Measurement.

As shown in Figure 1, the proposed measurement method is primarily based on sub-aperture stitching technology [21] and oblique-incidence laser interferometry [22]. Its core component is a horizontal laser interferometer, which serves as the fundamental measurement instrument. By integrating an additional reflective mirror, the system enables oblique-incidence measurements. A single-axis translation mechanism is employed to move the workpiece, facilitating sub-aperture stitching for large-area surface measurement.

First, based on the dimensions of the measured surface and the aperture size of the interferometer, the required number of sub-apertures and the optimal angle of oblique incidence are precisely calculated. This step ensures the completeness andw3 accuracy of the measurement. Subsequently, an appropriate stitching path is planned, and the oblique incidence angle is adjusted accordingly. Interference fringe data are then sequentially acquired at each sub-aperture position using the oblique-incidence measurement approach. These fringe patterns serve as the foundation for subsequent phase information retrieval. Once the interference data are obtained, the phase information is extracted using the Phase Shifting Interferometry (PSI) algorithm in combination with a phase unwrapping algorithm [23]. This step is critical to the measurement process, as it enables the conversion of fringe patterns into surface height data. It is worth noting that both the PSI and phase unwrapping algorithms are widely applied in optical metrology and are known for their ability to enhance measurement precision and stability. After acquiring the data for all sub-apertures, sub-aperture stitching is performed to integrate the local measurements into a complete surface dataset. This process enables the transition from local to global surface characterization. Finally, the compressed surface profile data along the longitudinal direction are reconstructed to recover the full surface topography. This final step achieves the overall objective of the measurement, providing a comprehensive and accurate surface profile of the measured component.

### 2.1. Principle of Oblique-Incidence Laser Interferometric Measurement

#### 2.1.1. Method of Oblique-Incidence Laser Interferometric Measurement

In conventional laser interferometric surface measurement, the measurement beam is incident perpendicular or nearly perpendicular to the test surface. Due to subtle variations in the surface topography—such as local height differences—optical path differences (OPDs) are introduced during beam propagation. These OPDs result in the formation of interference fringes when the measurement beam is combined with a reference beam on the reference surface.

**Figure 2:**
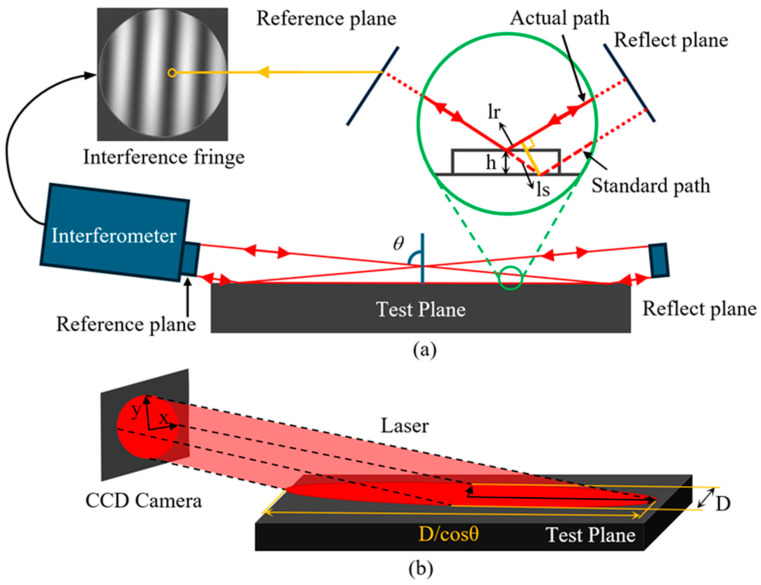
Principle of oblique-incidence laser interferometric measurement. (**a**) Principle of fringe formation; (**b**) Measurement range.

In contrast, the process of oblique-incidence laser interferometric measurement is more complex. As illustrated in Figure 2a, the measurement beam is projected onto the test surface at an incidence angle θ, forming an elliptical measurement footprint. The reflected beam from the test surface is subsequently directed to a mirror, which then reflects the beam back to the reference surface along its original path. As shown in Figure 2b, the resulting elliptical measurement area—with a minor axis equal to the interferometer aperture D, a major axis of D/cos(θ), and a total area of $S=D^{2}\pi /\left(4\cos\theta \right)$—is ultimately compressed into a circular fringe pattern of diameter D on the interference image. During this process, the measurement beam interacts with the test surface twice. Variations in the surface profile cause differences in the optical path between the test and reference beams, generating an optical path difference d at each corresponding position, as illustrated in Figure 2a. According to Equation (1), the relationship between the surface height difference h and the optical path difference d can be expressed as Equation (2). Furthermore, the relationship between the optical path difference and the interference phase leads to an expression linking the surface height h and the phase difference Δφ, as shown in Equation (3).

(1) $$\left\{\begin{array}{l} d=2(ls-lr)\cdot n_{0}\\ lr=\cos(180-2\theta )\cdot ls\\ ls=h/\cos\theta \end{array}\right.$$

Therefore,

(2) $$d=\frac{1}{4\cos\theta }\cdot \frac{1}{n_{0}}\cdot h$$

(3) $$h=\frac{1}{4\cos\theta }\cdot \frac{\lambda_{0}}{2\pi n_{0}}\cdot ∆\phi$$

where d is the optical path difference (OPD) between the actual beam path and the nominal reference path; *h* is the height deviation between the measured point and the nominal reference surface; *n*_0_ is the refractive index of the gas in the interferometric cavity; *l_r_* and *l_s_* represent the geometric lengths of the actual and nominal beam paths, respectively, that contribute to the optical path difference.

The phase-shifting interferometer obtains nine fringe intensity images with successive phase differences of 90° by using a piezoelectric transducer (PZT). These images are processed using the Phase-Shifting Interferometry (PSI) algorithm and a phase unwrapping algorithm [24], yielding phase difference data at all sampling positions. Based on the oblique incidence factor determined by the incident angle, the absolute surface height profile of the measured surface as Equation (4):

(4) $$M_{h}=\frac{1}{4\cos\theta }\cdot \frac{\lambda_{0}}{2\pi n_{0}}\cdot M_{∆\phi }$$

where *M_h_* denotes the height data of each point on the measured surface, *λ*_0_ is the laser wavelength, *n*_0_ is the refractive index of the interferometric cavity, and $M_{∆\emptyset }$ represents the phase difference at each point obtained through the PSI and phase unwrapping algorithms.

#### 2.1.2. Influence of the Incidence Angle on the Reflectivity of the Measured Surface in Oblique

The two coherent beams that generate interference fringes are the reference beam reflected by the reference surface and the measurement beam reflected back to the reference mirror. For rough surfaces, under normal incidence, most of the measurement beam will be scattered, resulting in a decrease in the intensity of the measurement beam and ultimately a reduction in the contrast of the interference fringes. As a result, the acquired interferogram is not clear enough, making it difficult for subsequent Phase Shifting Interferometry (PSI) and phase unwrapping algorithms to extract accurate phase information from the grayscale values. In practical engineering applications, when using a vertical incidence plane laser interferometer for high-precision flatness detection, the method to reduce astigmatism is to perform optical polishing on the product to reduce the surface roughness of the measured surface and improve the product’s reflectivity of the measurement beam. However, the oblique incidence interference stitching detection method proposed in this paper does not require polishing the product surface. According to the Fresnel equations shown in Equation (5), a relationship between reflectivity and the incident angle θ can be established: the larger the incident angle, the higher the reflectivity. This enhances the intensity of the desired interference signal relative to the scattered noise, thereby increasing the contrast of the interference fringes.

(5) $$R=\frac{R_{s}+R_{p}}{2}=\frac{1}{2}\left[{\left|\frac{n_{0}\cos\theta -N_{1}\sqrt{1-{\left(\frac{n_{0}}{N_{1}}\sin\theta \right)}^{2}}}{n_{0}\cos\theta +N_{1}\sqrt{1-{\left(\frac{n_{0}}{N_{1}}\sin\theta \right)}^{2}}}\right|}^{2}+{\left|\frac{n_{0}\sqrt{1-{\left(\frac{n_{0}}{N_{1}}\sin\theta \right)}^{2}}-N_{1}\cos\theta }{n_{0}\sqrt{1-{\left(\frac{n_{0}}{N_{1}}\sin\theta \right)}^{2}}+N_{1}\cos\theta }\right|}^{2}\right]$$

Of which $n_{0}$ represents the refractive index of the incident medium, and $N_{1}$ represents the refractive index of the measured reflecting surface. If the measured material is a light-absorbing material, then $N_{1}$ is expressed as $N_{1}=n_{1}-ki$, where k represents the extinction coefficient. When the incident angle *θ* is equal to zero—that is, under normal incidence—the baseline reflectivity $R_{0}={\left|(n_{0}-N_{1})/(n_{0}+N_{1})\right|}^{2}$ can be obtained.

**Figure 3:**
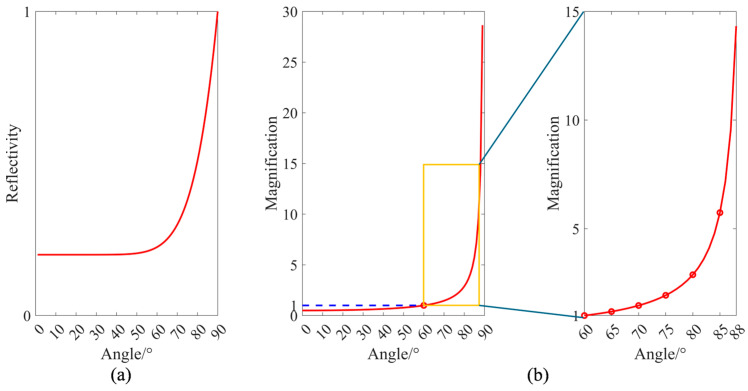
Influence of the oblique incidence angle: (**a**) Relationship between incidence angle and surface reflectance; (**b**) Relationship between incidence angle and error magnification factor.

Using the Schlick approximation:

(6) $$R_{\left(T,\theta \right)}=R_{0}+\left(1-R_{0}\right){\left(1-\cos\theta \right)}^{5}$$

of which $R_{\left(T,\theta \right)}$ denotes the reflectivity of the test surface at an incident angle *θ*, the reflectivity of the test surface under oblique incidence can be derived from the baseline reflectivity and the incident angle. As shown in Figure 3a, the reflectivity of the test surface increases significantly with the incident angle in the range of 80° to 90°.

#### 2.1.3. Error Analysis of Oblique Incidence Laser Interferometric Measurement

The oblique-incidence laser interferometry method significantly extends the effective measurement range compared to conventional normal-incidence plane interferometry. However, this extension introduces two primary sources of systematic error: (1) mirror-induced system error, which arises from the amplified surface figure deviations of the reference and return mirrors [25]; and (2) spatial resolution degradation along the ellipse’s major axis.

Mirror-induced system error also exists in conventional normal-incidence laser interferometry and is typically embedded within the phase map obtained by the interferometer. However, since the reference mirror generally exhibits a surface flatness much higher than that of the test surface, its influence is often negligible. In oblique-incidence laser interferometry, by contrast, the phase error arises from both the reference mirror and the return mirror, as shown in Equation (7), of which $M_{∆\phi }$ means the phase difference data corresponding to all measurement pixels within the measurement range, $M_{T\phi }$ means the phase difference data generated by the measured surface, $M_{R\phi }$ means the phase difference data generated by the reference surface, $M_{F\phi }$ means the phase difference data generated by the reflecting surface. When the incidence angle exceeds 60°, the obliquity magnification factor significantly amplifies mirror-induced errors. Based on Equations (4) and (7), the overall mirror-induced system error, denoted as $Differ_{\phi }$, can thus be derived [26].

(7) $$M_{∆\phi }=M_{T\phi }+2M_{R\phi }+2M_{F\phi }$$

(8) $$Differ_{\phi }=2\cdot \frac{1}{4\cos\theta }\cdot \frac{\lambda_{0}}{2\pi n_{0}}\cdot \left(M_{R\phi }+M_{F\phi }\right)$$

As shown in Figure 3b, when the incidence angle exceeds 80°, the magnification factor increases sharply, introducing significant wavefront system errors that affect the final measurement results. Furthermore, during the subsequent sub-aperture stitching process, sub-apertures are aligned and merged based on the slope of the overlapping regions on the test surface. Consequently, the slope characteristics of the error matrix are inherently incorporated into each sub-aperture, leading to a tilted compensation surface during stitching and resulting in stitching-induced errors.

The spatial resolution loss error arises from the projection effect in oblique incidence interferometry, where the originally elliptical wavefront region on the test surface is compressed into a circular wavefront of diameter *D*. In conventional normal-incidence measurements, each CCD pixel corresponds to a square sampling area with a resolution of d×d. However, under oblique incidence, this sampling area becomes elongated in one direction, resulting in an effective resolution of $d\times \left(1/\cos\theta \times d\right)$. This resolution degradation leads to local averaging of the wavefront data: regions originally represented by 1/cosθ pixels are merged into fewer data points, causing peaks to be suppressed and valleys to rise. Consequently, the peak-to-valley (PV) value of the measured surface tends to decrease, while the root-mean-square (RMS) deviation is relatively unaffected.

### 2.2. Principle of Sub-Aperture Stitching Measurement

The sub-aperture stitching measurement method consists of three main components: single-aperture measurement, stitching path planning, and stitching algorithm. The single-aperture measurement step involves selecting an appropriate method for acquiring point cloud data of the surface, such as white-light interferometry, spherical laser interferometry, or plane laser interferometry. The choice of measurement technique depends on the surface characteristics of the object under test and the required measurement accuracy. In this process, the surface profiles acquired from each sub-aperture are independent and typically contain errors such as lateral displacement and tilt.

To obtain full-aperture data, it is necessary to unify the coordinate systems of the sub-apertures through stitching path planning. The selection of the stitching path depends on the surface geometry of the object under test and must ensure sufficient overlap between adjacent sub-aperture measurement regions. For circular surfaces, a concentric path strategy can be adopted, whereas for rectangular surfaces, a grid-based row-column path is preferred to minimize the number of stitching operations. Ultimately, a global surface map covering all sub-apertures is generated based on the aperture configuration and the selected stitching path [21].

On the global surface map, the overlapping regions between sub-apertures are identified based on their aperture size and spatial coordinates. A stitching algorithm is then applied to calculate the relative translation and tilt between sub-apertures, and the data are merged after compensation.

#### 2.2.1. Sub-Aperture Stitching Algorithm

The primary objective of the sub-aperture stitching algorithm is to align the slope and positional deviations of data acquired at different locations. To ensure reliable registration, each sub-aperture must share an overlapping region with its adjacent sub-apertures, covering no less than one-quarter of the aperture area [27].

Each sub-aperture is assigned an index based on the measurement path. The measurement data from the sub-aperture with index 1 is used as the reference to establish a global coordinate system. In this coordinate system, the relationship between the measured surface data $Z_{i}(x,y)$ of each sub-aperture and the ideally stitched data ${Z_{i}}^{\prime }(x,y)$ is given by the following Equation (9):

(9) $${Z_{i}}^{\prime }(x,y)=Z_{i}(x+x_{i},y+y_{i})+a_{i}x+b_{i}y+c_{i}$$

of which ${Z_{i}}^{\prime }(x,y)$ represents the ideally stitched data after compensation; $Z_{i}(x,y)$ is the measured surface profile data from the sub-aperture with index *i*; $x_{i},y_{i}$ are the coordinates of the i-th sub-aperture data; $a_{i}x+b_{i}y+c_{i}$ is the compensation function for slope and translation relative to the reference sub-aperture, where $a_{i}$ and $b_{i}$ are the tilt coefficients in the *X* and *Y* directions, respectively, and $c_{i}$ is the translation coefficient in the *Z* direction.

**Figure 4:**
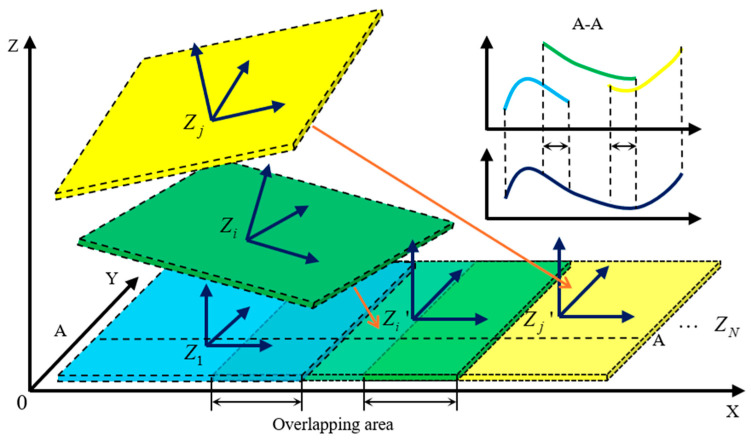
Schematic diagram of the sub-aperture stitching measurement principle.

As illustrated in Figure 4, the stitching process involves compensating for the displacement along the X, Y, Z-axis and the tilts along the X and Y axes in the overlapping regions of the sub-aperture data. The displacement along the X and Y axes corresponds to the motion of the translation stage and can be determined from the mechanical coordinates provided by the stage’s displacement sensors. The compensation for the Z-axis displacement and angular tilts along the X and Y axes is performed using a global optimization algorithm based on the least squares method, as given in Equation (10) [21]:

(10) $$V=\sum_{i=1}^{N}\sum_{j=i}^{N}{\left[\left(Z_{i}(x+x_{i},y+y_{i})+a_{i}x+b_{i}y+c_{i}\right)-\left(Z_{j}(x+x_{j},y+y_{j})+a_{j}x+b_{j}y+c_{j}\right)\right]}^{2}$$

of which $Z_{i}(x,y)$ and $Z_{j}(x,y)$ represent the measured surface profile data of two arbitrary sub-apertures, while $a_{i}x+b_{i}y+c_{i}$ and $a_{j}x+b_{j}y+c_{j}$ denote their respective compensation functions. Based on the least squares method, the slope compensation functions for each sub-aperture are obtained by solving for the parameters $a_{i}(i=1\ldots N)$, $b_{i}(i=1\ldots N)$, and $c_{i}(i=1\ldots N)$. Taking the partial derivatives of Equation (10) and setting them to zero yields:

(11) $$S_{i}=\left\{\begin{array}{c} \frac{\partial V_{i}}{\partial a_{i}}\\ \frac{\partial V_{i}}{\partial b_{i}}\\ \frac{\partial V_{i}}{\partial c_{i}} \end{array}\right.=\left\{\begin{array}{c} \sum_{j=1}^{N}2\left[Z_{ij}(x,y)+a_{ij}x+b_{ij}y+c_{ij}\right]x\\ \sum_{j=1}^{N}2\left[Z_{ij}(x,y)+a_{ij}x+b_{ij}y+c_{ij}\right]y\\ \sum_{j=1}^{N}2\left[Z_{ij}(x,y)+a_{ij}x+b_{ij}y+c_{ij}\right] \end{array}\right.=0$$

of which $S_{i}$ denotes the partial derivatives of V with respect to $a_{i},\;{\;b}_{i}\;and\;c_{i}$; $Z_{ij}$ represents the difference in the overlapping region between $Z_{i}$ and $Z_{j}$; $a_{ij}=a_{i}-a_{j}$; $b_{ij}\;$= $b_{i}-b_{j}$; and $c_{ij}$=$\;c_{i}-c_{j}$. By simplifying the expression into matrix form, Equation (12) can be obtained:

(12) $$\left[\begin{array}{ccc} x^{2} & xy & x\\ xy & y^{2} & y\\ x & y & 1 \end{array}\right]\cdot \left[\begin{array}{c} \sum_{j=1}^{N}a_{ij}\\ \sum_{j=1}^{N}b_{ij}\\ \sum_{j=1}^{N}c_{ij} \end{array}\right]=-\left[\begin{array}{c} \sum_{j=1}^{N}Z_{ij}(x,y)\cdot x\\ \sum_{j=1}^{N}Z_{ij}(x,y)\cdot y\\ \sum_{j=1}^{N}Z_{ij}(x,y) \end{array}\right]$$

By applying the above derivation to the surface profile fitting process, we obtain Equation (13):

(13) $$\sum_{j=1}^{N}\left[\begin{array}{ccc} X*X*Mask_{ij} & Y*X*Mask_{ij} & X*Mask_{ij}\\ X*Y*Mask_{ij} & Y*Y*Mask_{ij} & Y*Mask_{ij}\\ X*Mask_{ij} & Y*Mask_{ij} & J*Mask_{ij} \end{array}\right]\left[\begin{array}{c} a_{ij}\\ b_{ij}\\ c_{ij} \end{array}\right]=-\sum_{j=1}^{N}\left[\begin{array}{c} Z_{ij}*X\\ Z_{ij}*Y\\ Z_{ij} \end{array}\right]$$

where *X* and *Y* denote the horizontal and vertical coordinate matrices, respectively, and $Mask_{ij}$ represents the mask matrix of the overlapping region between $Z_{i}$ and $Z_{j}$, with values of 1 in the overlapping region and 0 elsewhere. The above equation can be simplified as:

(14) $$\sum_{j=1}^{N}Q_{ij}\left[\begin{array}{c} a_{ij}\\ b_{ij}\\ c_{ij} \end{array}\right]=-\sum_{j=1}^{N}P_{ij}$$

of which

$$Q_{ij}=\left[\begin{array}{ccc} X*X*Mask_{ij} & Y*X*Mask_{ij} & X*Mask_{ij}\\ X*Y*Mask_{ij} & Y*Y*Mask_{ij} & Y*Mask_{ij}\\ X*Mask_{ij} & Y*Mask_{ij} & J*Mask_{ij} \end{array}\right],\;P_{ij}=-\left[\begin{array}{c} Z_{ij}*X\\ Z_{ij}*Y\\ Z_{ij} \end{array}\right]$$

In order to compute $a_{i},\;b_{i}$ and $c_{i}$, we define:

$$k_{i}=\left[\begin{array}{c} a_{i}\\ b_{i}\\ c_{i} \end{array}\right],\;K=\left[\begin{array}{c} k_{1}\\ k_{2}\\ k_{3}\\ .\\ .\\ .\\ k_{N} \end{array}\right]$$

To construct the matrix equation:

(15) AK=B

of which

$$A=\left[\begin{array}{ccccc} \sum_{j=1}^{N}Q_{1j} & -Q_{12} & -Q_{13} & \ldots & -Q_{1N}\\ -Q_{21} & \sum_{j=1}^{N}Q_{2j} & -Q_{23} & \ldots & -Q_{2N}\\ -Q_{31} & -Q_{32} & \sum_{j=1}^{N}Q_{3j} & \ldots & \ldots \\ \ldots & \ldots & \ldots & \ldots & -Q_{N-1N}\\ -Q_{N1} & -Q_{N2} & \ldots & -Q_{NN-1} & \sum_{j=1}^{N}Q_{Nj} \end{array}\right],\;B=\left[\begin{array}{c} \sum_{j=1}^{N}P_{1j}\\ \sum_{j=1}^{N}P_{2j}\\ \sum_{j=1}^{N}P_{3j}\\ .\\ .\\ .\\ \sum_{j=1}^{N}P_{Nj} \end{array}\right]$$

Since sub-aperture No. 1 is chosen as the reference plane, its compensation coefficient vector is set as $k_{1}={\left[\begin{array}{ccc} 0 & 0 & 0 \end{array}\right]}^{T}$. By substituting matrices A and B into the objective function, the compensation coefficient vector K can be obtained by solving Equation (15), thus determining the compensation plane function for each sub-aperture. Once the slope terms are removed using these compensation functions, the complete surface profile can be accurately reconstructed by stitching the sub-aperture data. Each pixel measurement point within the overlapping regions typically contains data from two or more sub-apertures. After the processing, these data points may not be exactly consistent due to random measurement errors and wavefront systematic errors. We calculate the average of these data points and use this mean value as the final sub-aperture measurement result for that point.

#### 2.2.2. Path Planning for Sub-Aperture Stitching

**Figure 5:**
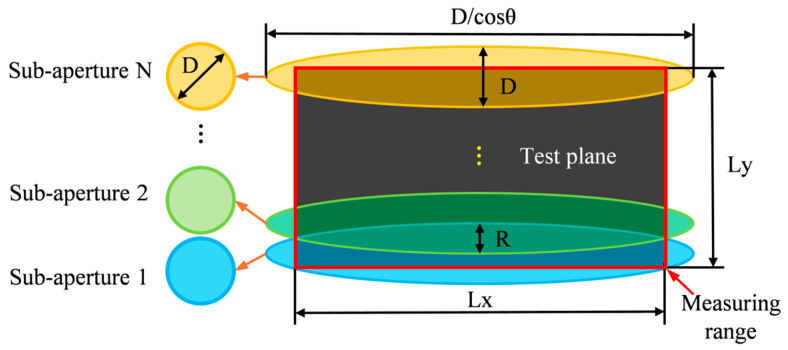
Illustration of the stitching and oblique incidence measurement path planning.

Different from classical sub-aperture planning, the actual measurement area of each sub-aperture in the proposed method is enlarged into an elliptical shape due to the oblique incidence measurement technique. Therefore, when planning the stitching paths, the actual elliptical measurement area should be considered as the effective measurement region for each sub-aperture when calculating the coverage, as shown in Figure 5. Additionally, it is essential to ensure that the overlapping region between adjacent sub-apertures contains a sufficient amount of data to participate in the stitching process, thereby ensuring stitching accuracy.

(16) $$N=\left\lceil Ly/l\right\rceil$$

(17) $$\theta =\cos^{-1}(\frac{\sqrt{D^{2}-l^{2}}}{Lx})$$

During the preparation of this manuscript, the authors used ChatGPT-4o (version: 2024.05) to enhance the coherence and accessibility of the Abstract and Summary sections. The authors have reviewed and edited the output and take full responsibility for the content of this publication.

## 3. Numerical Demonstration

### 3.1. Simulation Conditions

**Figure 6:**
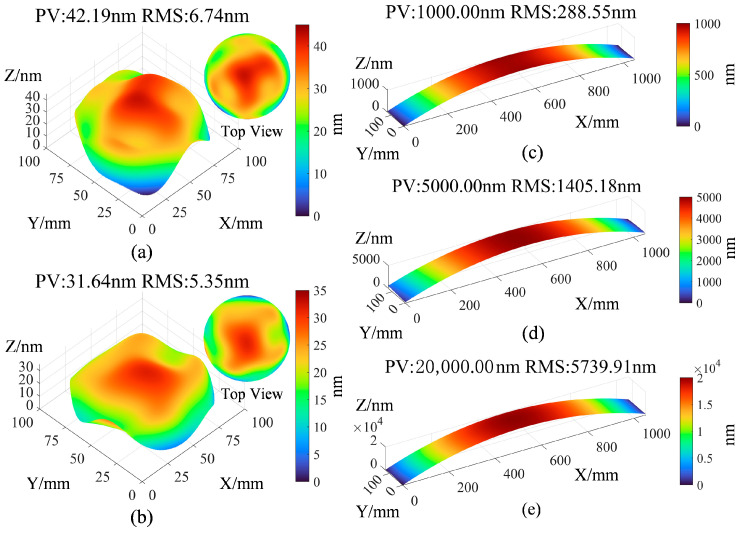
Simulation surface profiles of the measurement experiment (**a**) Reflective mirror surface Profile; (**b**) Reference mirror surface profile; (**c**) Long guide rail plane profile with PV = 1000 nm; (**d**) Long guide rail plane profile with PV = 5000 nm; (**e**) Long guide rail plane profile with PV = 20,000 nm.

To verify the theoretical effectiveness of the proposed method, a simulation model of the measurement experiment was established. Reflective and reference surfaces with a diameter of 100 mm were constructed using Zernike polynomials dominated by spherical terms, with PV values of 1/15λ and 1/20λ (λ = 632.8 nm), simulating the interferometer’s reference and reflective mirrors, as shown in Figure 6a,b. Additionally, three experimental planar surfaces with dimensions of 1050 mm in length and 130 mm in width were modeled using polynomials dominated by the X^2^ term, featuring flatness values of 1 µm, 5 µm, and 20 µm to represent different processing stages of high aspect ratio surfaces, as shown in Figure 6c–e.

### 3.2. Simulation Procedure

**Figure 7:**
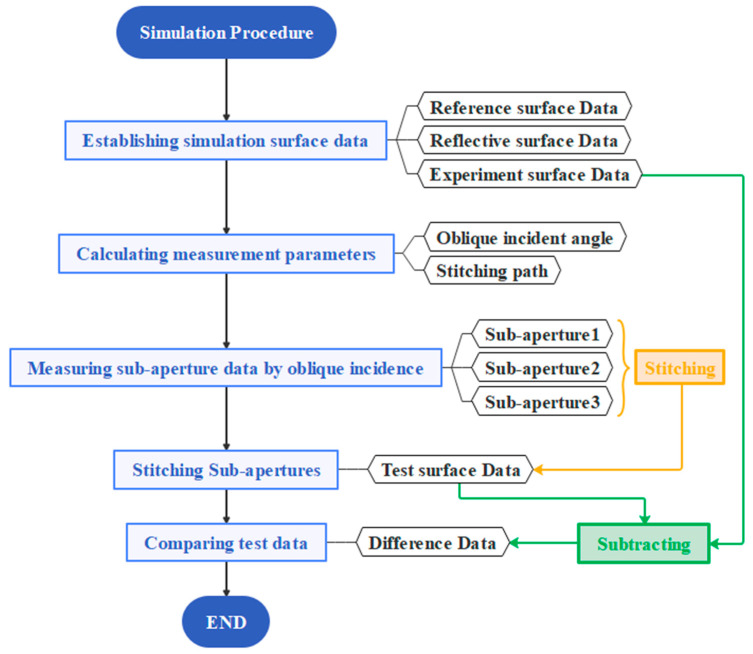
Simulation flowchart.

Based on the basic simulation parameters described in Section 3.1, the simulation process is divided into five main steps, as illustrated in Figure 7.

After defining the reference surface, reflective surface, and experimental surface data, the oblique incidence coefficient and the sub-aperture stitching path must be calculated according to the diameters of the reference and reflective surfaces, as well as the length and width of the test surface. The parameter l is set as D/2, and the calculation proceeds as follows:

(18) $$N=\left\lceil Ly/l\right\rceil =3$$

(19) $$\theta =\cos^{-1}(\frac{\sqrt{D^{2}-l^{2}}}{Lx})=85.2676{}^{\circ}$$

From the calculated sub-aperture coverage and the oblique incidence angle, the number of sub-apertures is determined to be *N* = 3. Next, the reference and reflective surfaces are adjusted so that the incident angle θ on the test surface reaches 85.2676°.

**Figure 8:**
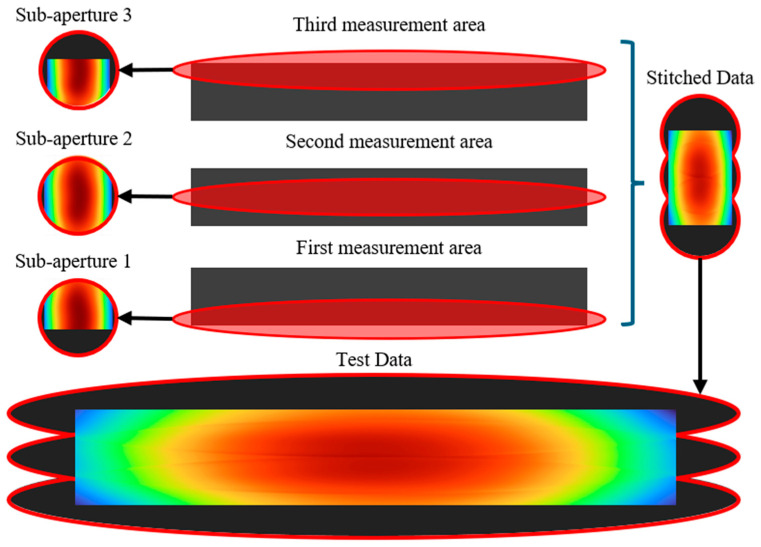
Illustration of the simulation process.

Following the pre-defined path planning, the oblique incidence simulation is used to acquire the wavefront phase data at each measurement position. The resulting sub-aperture phase maps—sub-aperture 1, 2, and 3—are shown in Figure 8.

Subsequently, the height profile data and spatial resolution of each sub-aperture are derived using the oblique incidence coefficient, interferometer wavelength, and refractive index, with the conversion described by the following equation:

(20) $$M_{h}=\frac{1}{4\cos\theta }\cdot \frac{\lambda_{0}}{2\pi n_{0}}\cdot M_{∆\phi }=3.0302\cdot \frac{632.8}{2\pi \cdot 1.00027}{\cdot M}_{∆\phi }=305.0989\cdot M_{∆\phi }$$

Subsequently, the three sub-aperture datasets are stitched together according to the predefined stitching path. Based on the spatial resolution, the actual-scale 3D point cloud data are reconstructed. Finally, to verify the measurement accuracy, the stitched surface profile is compared with the original surface profile to obtain the surface figure measurement error in the simulation.

### 3.3. Simulation Result

**Figure 9:**
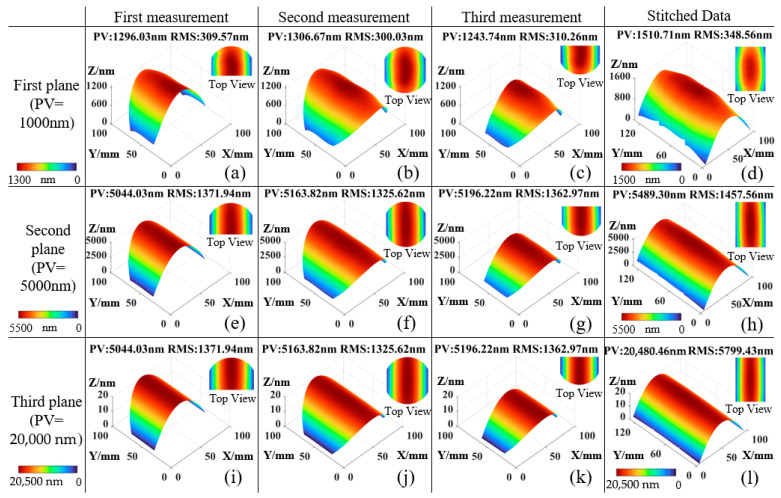
Simulation measurement results: (**a**) Sub-aperture measurement 1 of Surface 1; (**b**) Sub-aperture measurement 2 of Surface 1; (**c**) Sub-aperture measurement 3 of Surface 1; (**d**) Stitched measurement of Surface 1; (**e**) Sub-aperture measurement 1 of Surface 2; (**f**) Sub-aperture measurement 2 of Surface 2; (**g**) Sub-aperture measurement 3 of Surface 2; (**h**) Stitched measurement of Surface 2; (**i**) Sub-aperture measurement 1 of Surface 3; (**j**) Sub-aperture measurement 2 of Surface 3; (**k**) Sub-aperture measurement 3 of Surface 3; (**l**) Stitched measurement of Surface 3.

Based on the simulation procedure described above, measurement simulations were conducted on three surfaces with different flatness levels. The results are presented in Figure 9. Figure 9a–c show the three sub-aperture measurement results of Simulated Surface 1, and Figure 9d presents the stitched surface profile, with a measured flatness of PV = 1501.71 nm and RMS = 348.56 nm. Figure 9e–g correspond to the three sub-aperture measurements of Simulated Surface 2, and Figure 9h shows the stitched result, yielding PV = 5489.30 nm and RMS = 1457.56 nm. Figure 9i–k display the three sub-aperture measurements of Simulated Surface 3, while Figure 9l shows the stitched surface profile, with PV = 20,480.46 nm and RMS = 5799.43 nm.

(21) $$\left\{\begin{array}{c} x=\frac{1}{cos\theta }\cdot x0=12.12\cdot x0\\ y=y0 \end{array}\right.$$

**Figure 10:**
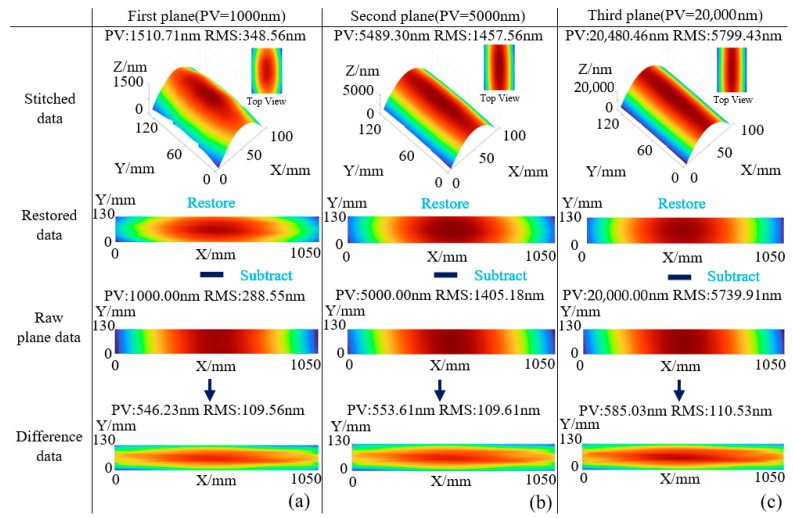
Simulation measurement error analysis. (**a**) Error calculation process for measured Surface 1; (**b**) Error calculation process for measured Surface 2; (**c**) Error calculation process for measured Surface 3.

According to Equation (21), the spatial resolutions in the X and Y directions are calculated, allowing each data point to be mapped to its corresponding planar coordinate. This enables the reconstruction of the measurement data map in actual dimensions. Subsequently, the simulated measurement results of the three surfaces are subtracted from their respective original surface profiles to obtain the measurement error distributions, as shown in Figure 10. Figure 10a illustrates the data reconstruction and comparison process for Surface 1, with a resulting error surface profile of PV = 546.23 nm and RMS = 109.56 nm. Figure 10b shows the same process for Surface 2, yielding PV = 553.61 nm and RMS = 109.61 nm. Figure 10c presents the results for Surface 3, with PV = 583.03 nm and RMS = 110.53 nm. The similarity in error values across surfaces with different flatness levels suggests that the measurement errors primarily originate from the mirror-induced system error and the spatial resolution loss described in Section 2.1.

**Figure 11:**
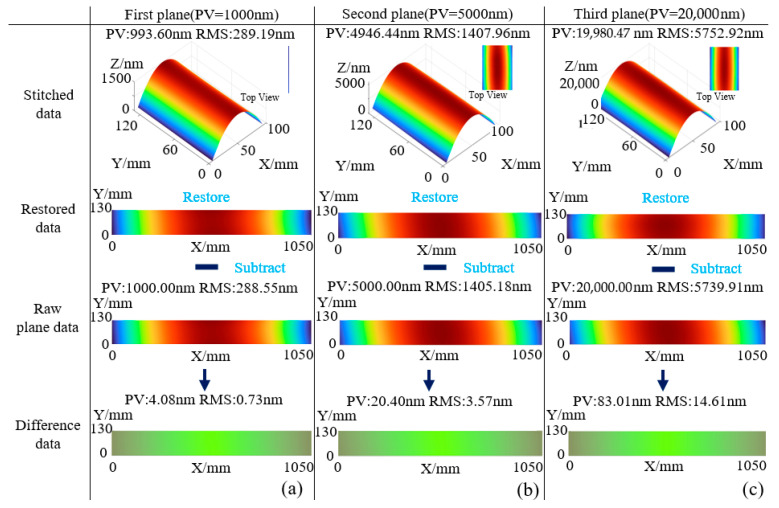
Simulated measurement error with ideal planar mirrors: (**a**) Measurement error analysis for Surface 1; (**b**) Measurement error analysis for Surface 2; (**c**) Measurement error analysis for Surface 3.

To verify the source of the measurement error, the reference and reflective mirrors were set as ideal planes (PV = 0, RMS = 0), and the simulation was repeated. The results are shown in Figure 11. Figure 11a shows the data reconstruction and comparison process for Surface 1, where the resulting error surface profile yields PV = 4.08 nm and RMS = 0.73 nm. Figure 11b shows the same process for Surface 2, with PV = 20.40 nm and RMS = 3.57 nm. Figure 11c presents the results for Surface 3, with PV = 83.01 nm and RMS = 14.61 nm. When both the reference and reflective surfaces are ideal, the flatness errors of the reconstructed surfaces are significantly reduced, with PV values representing only approximately 0.4% of the original surfaces and RMS values around 0.25%. This clearly demonstrates the influence of the reference and reflective mirror surface profiles on the measurement accuracy.

The validity of the proposed oblique-incidence sub-aperture stitching method was verified through numerical simulation. Surface profiles with flatness values of 1 μm, 5 μm, and 20 μm were reconstructed, and the post-stitched results (PV and RMS) exhibited high consistency with the original surfaces, demonstrating the method’s capability in accurately reproducing the topography of high aspect ratio surfaces.

When non-ideal reference and return mirrors (with PV errors of 1/20λ and 1/15λ, respectively) were used, the resulting measurement deviations (PV ≈ 546–583 nm; RMS ≈ 109–110 nm) were found to be largely independent of the surface flatness. These errors are primarily attributed to system-level aberrations introduced by the mirror figures. This part of the error is also a cause of measurement artifacts in the measurement results. In practice, measurement artifacts can be reduced by using high-precision reference mirrors and reflectors, or by adopting absolute measurement methods such as the three-flat test, but the latter has strict requirements for the laboratory environment.

In contrast, when ideal reference and return mirrors (PV = 0, RMS = 0) were employed, the measurement deviations were significantly reduced. The residual errors exhibited proportionality to the original surface flatness, with PV deviations approximately 0.4% and RMS deviations approximately 0.25% of the true values. This suggests that the remaining errors are dominated by resolution loss and intrinsic model limitations.

**Remark** **1.**

1. *Surface figure errors of the reference and return mirrors constitute the primary source of systematic error, and their influence is independent of the surface under test.*
2. *Even in the absence of mirror figure errors, minor deviations persist and scale proportionally with surface flatness, likely due to spatial resolution limitations inherent to the reconstruction algorithm.*

## 4. Measurement Experiment

To further validate the effectiveness of the algorithm and conduct flatness measurement experiments on discontinuous surfaces, this paper proposes an experimental method using a light-blocking mask. In employed to assist in the process.

### 4.1. Experiment Setup

**Figure 12:**
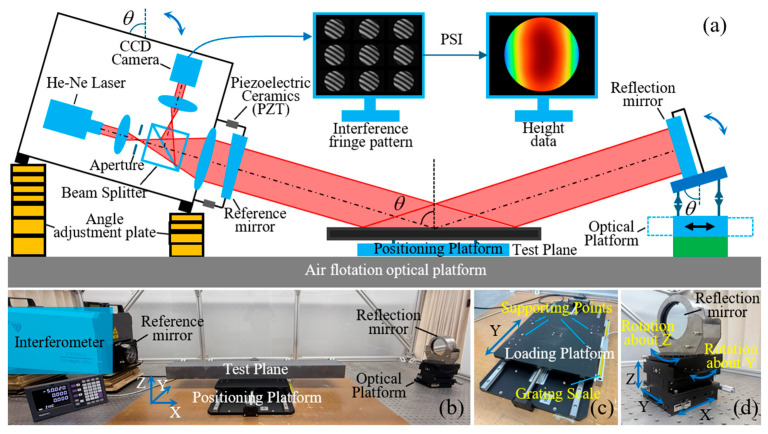
Illustration of the experimental setup: (**a**) schematic diagram of the setup; (**b**) photograph of the experimental system; (**c**) photograph of the translation stage; (**d**) photograph of the return mirror.

The experimental setup, as illustrated in Figure 12a,b, primarily consists of a 4-inch phase-shifting laser interferometer, a 100 mm aperture return mirror, an angle-adjustable flat plate, a single-axis translation stage, and a five-axis optical adjustment platform. As shown in Figure 12c, the single-axis translation stage enables movement of the sample holder along the Y-axis with a travel range of 500 mm, and the displacement is monitored by a linear encoder. The return mirror, depicted in Figure 12d, is mounted on a five-axis optical platform that allows translational adjustment along the X, Y, and Z axes, as well as rotational alignment about the Y and Z axes. This configuration ensures precise alignment of the mirror’s central position and reflection angle.

### 4.2. Validation Experiment

To compare the conventional vertical-incidence sub-aperture stitching measurement with the oblique-incidence stitching method proposed in this study, a validation experiment was conducted. The test object was the surface of a SiC ceramic guideway with a length of 1050 mm and a width of 130 mm. The surface roughness of this specimen was non-uniform: only the central 700 mm region had been polished, while both ends remained in a ground, unpolished state. Two measurements were performed on this surface: one using the proposed oblique-incidence stitching method, and the other using a conventional sub-aperture stitching approach based on vertical incidence.

**Figure 13:**
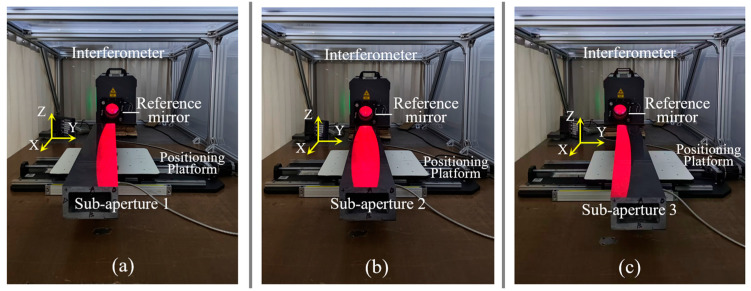
Physical photos of the experimental measurement process: (**a**) Measurement of Sub-aperture 1; (**b**) Measurement of Sub-aperture 2; (**c**) Measurement of Sub-aperture 3.

**Figure 14:**
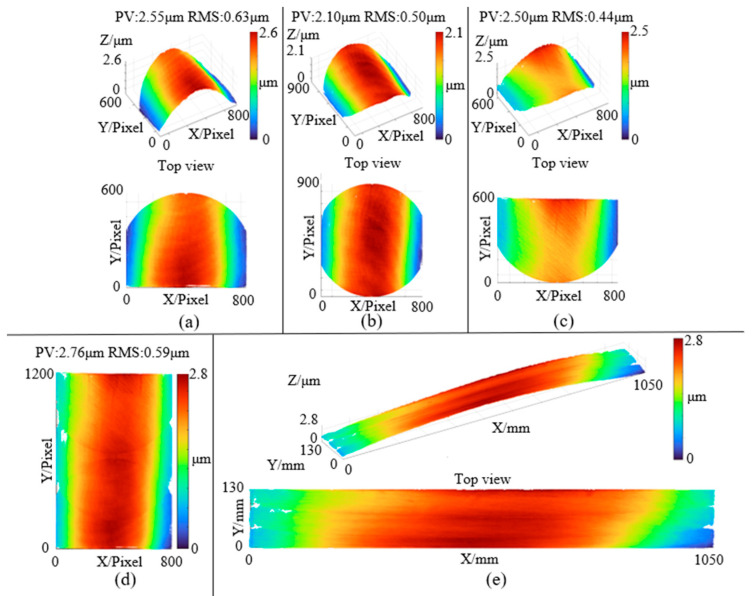
Measurement data: (**a**) Sub-aperture 1 measurement data; (**b**) Sub-aperture 2 measurement data; (**c**) Sub-aperture 3 measurement data; (**d**) Stitched data; (**e**) Final measurement result data.

For the experimental group, the measurement procedure followed the same steps as the simulation process. Experimental parameters—including the incident angle, sub-aperture center spacing, and spatial resolution—were set in accordance with the simulation settings. The actual measurement process is shown in Figure 13a–c, where the red regions indicate the positions of the three sub-aperture measurements. The results of the three measurements are presented in Figure 14. Figure 14a shows the height data from Sub-aperture 1 measured using the oblique-incidence method (X and Y axes represent pixel coordinates), with a PV of 2.55 μm and an RMS of 0.63 μm. Figure 14b shows the height data from Sub-aperture 2, with a PV of 2.10 μm and an RMS of 0.50 μm. Figure 14c shows the height data from Sub-aperture 3, with a PV of 2.50 μm and an RMS of 0.44 μm. Figure 14d shows the stitched full-aperture height data of the guideway (in pixel coordinates), with a PV of 2.76 μm and an RMS of 0.59 μm. The actual-scale data, reconstructed based on the spatial resolution, are shown in Figure 14e.

**Figure 15:**
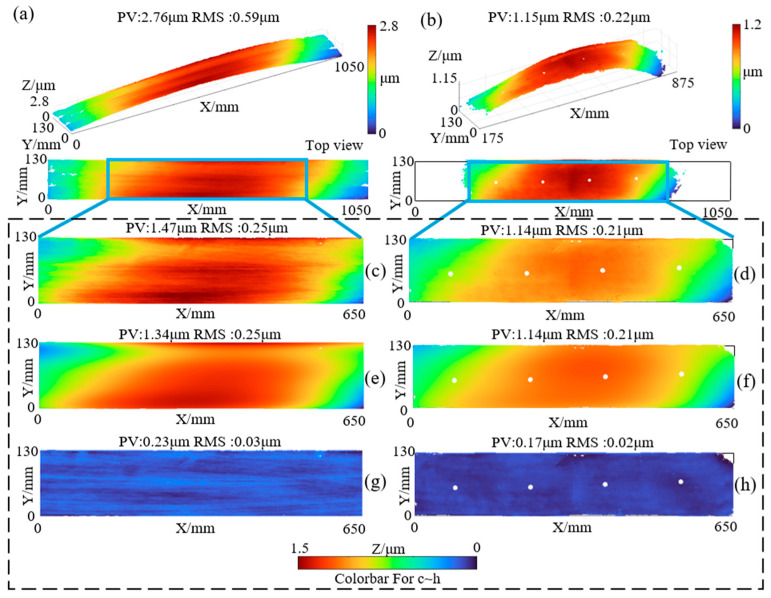
Comparison of measurement results: (**a**) Slant-incidence sub-aperture stitching measurement data (experimental group); (**b**) Traditional sub-aperture stitching measurement data (control group); (**c**) Extracted measurement data from the experimental group; (**d**) Extracted measurement data from the control group; (**e**) Fifth-order polynomial fitting of extracted data from the experimental group; (**f**) Fifth-order polynomial fitting of extracted data from the control group; (**g**) Residuals of polynomial fitting for the experimental group; (**h**) Residuals of polynomial fitting for the control group.

For the control group, a 300 mm-aperture interferometer was used to perform sub-aperture stitching measurements on the experimental surface. The measurement results are shown in Figure 15b. In Figure 15b there are 4 obvious prototype data missing points, which are caused by the matte marker points used for the alignment of the mechanical coordinate system in sub-aperture stitching. The number of these missing data points is less than 5% of all data points and will not have a significant impact on the comparative data. Compared with the proposed method in this study, this approach could only measure the polished region of the surface, while the unpolished areas failed to yield valid measurement data. Consequently, the final measurement result covered only a 700 mm × 130 mm region with surface flatness values of PV: 1.15 μm and RMS: 0.22 μm. In contrast, the oblique incidence method proposed in this paper exhibits superior adaptability to surface roughness. As visualized in Figure 15a, it can effectively capture interference fringe data from rough surfaces unaffected by uneven roughness, a capability lacking in the traditional laser interferometry used in the control group. This fundamental difference arises because the traditional method’s performance is heavily dependent on interference fringe contrast, which deteriorates significantly for materials like SiC ceramics with high roughness (and thus low reflectivity), rendering the PSI algorithm unable to compute wavefront phase data. Meanwhile, the proposed method overcomes such limitations. To verify the consistency between the two methods, height data were extracted from the same 650 mm × 130 mm region of the surface measured by both methods. The extracted data from the experimental group are shown in Figure 15c with surface flatness of PV: 1.47 μm and RMS: 0.25 μm, while those from the control group are shown in Figure 15d with PV: 1.14 μm and RMS: 0.21 μm.

Due to differing spatial resolutions between the two data sets, direct subtraction was infeasible. Therefore, polynomial fitting was applied to both extracted data sets, and only the results after fifth-order polynomial fitting were compared, as shown in Figure 15e,f. Figure 15g,h present the residuals between the measured data and fitted data for the experimental and control groups, respectively. It should be noted that lower-order (third-order) fitting fails to capture subtle surface changes, resulting in large residuals. In practical engineering analysis, higher-order polynomial surfaces have minimal impact on processing feedback, as they primarily consist of random errors from the measurement process (such as those caused by vibration and air disturbance, which affect the measuring beam’s optical path and cannot be eliminated by the Phase Shifting Interferometry (PSI) algorithm) and stitching system errors (arising from wavefront system errors introduced by the surface shapes of reference and reflecting mirrors, leading to measurement artifacts in stitching overlap areas that are difficult to compensate for). Therefore, if residuals do not interfere with processing feedback on the measurement target, they can satisfy the requirements of comparative analysis.

**Figure 16:**
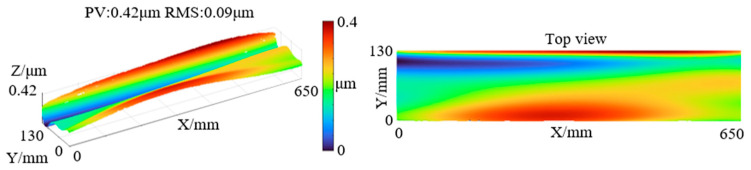
Fifth-order polynomial coefficient difference fitting data of the two datasets.

Subsequently, the fifth-order polynomial coefficients obtained from the experimental and control groups were subtracted to derive the coefficient differences. Using these coefficient differences, the measurement height difference between the two experiments was reconstructed, as shown in Figure 16, with PV: 0.42 μm and RMS: 0.09 μm.

In summary, the PV difference between the experimental and control measurements was 0.33 μm, and the RMS difference was 0.04 μm, while the fitted difference surface exhibited PV of 0.42 μm and RMS of 0.09 μm, consistent with the simulation results. Furthermore, for the grinding and polishing stages, the proposed measurement system fully satisfies the micron-level flatness measurement requirements.

### 4.3. Experiment on Flatness Convergence of Ceramic Guideway

**Figure 17:**
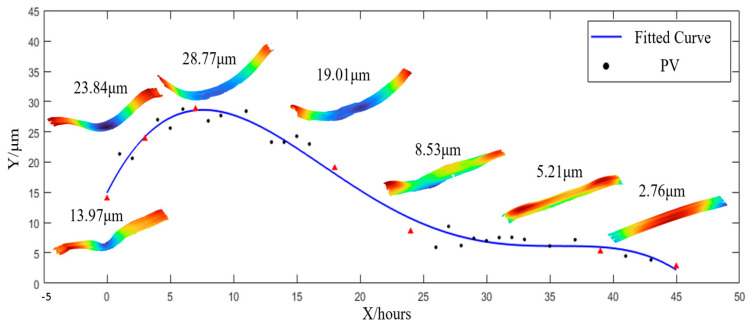
Flatness convergence curve of the 1050 mm guideway. Red arrow: variation trend.

After validating the effectiveness of the proposed method in Section 4.2, the oblique-incidence stitching interferometric technique was further applied to demonstrate its significance in monitoring surface figure convergence during the grinding process of a SiC ceramic guideway. It was applied to monitor the flatness during the lapping process of a SiC ceramic guideway surface, providing rapid feedback for the machining process. The measured surface size was 1050 mm × 130 mm, and the grinding abrasive used was diamond particles with a W10 grit size. As shown in Figure 17, the initial surface condition corresponded to that after diamond wheel grinding, with a PV of 13.97 μm. The measurement interval was set to every 1–2 h of grinding, with a total lapping duration of 39 h. During the first 7 h, the process mainly involved removing grinding marks, as the workpiece surface gradually aligned with the grinder surface and the grinding marks progressively disappeared. Subsequently, the flatness of the workpiece surface began to converge, stabilizing after approximately 35 h of processing, ultimately achieving a flatness of 2.76 μm.

### 4.4. Experimental Summary

The validation experiments demonstrate that the proposed oblique-incidence stitching interferometric measurement method can effectively measure large-scale surfaces with non-uniform roughness, such as SiC ceramic guideways, including both polished and unpolished regions. In contrast, the traditional perpendicular-incidence sub-aperture stitching interferometric method can only measure the polished region (700 mm range). The oblique-incidence method yields a complete surface measurement (1050 mm × 130 mm) with a PV value of 2.76 μm and an RMS value of 0.59 μm, showing good measurement consistency with the traditional method in the overlapping polished region (650 mm × 130 mm), where the PV deviation is 0.33 μm and the RMS deviation is 0.04 μm. Furthermore, comparison based on fifth-order polynomial fitting reveals minimal measurement differences between the two methods (fitted difference surface PV: 0.42 μm, RMS: 0.09 μm), confirming the reliability of the proposed approach.

The practical application experiment monitoring the surface grinding process of the SiC ceramic guideway over 39 h further verifies the method’s effectiveness. The measurement results clearly capture the surface evolution from the initial post-grinding state (PV: 13.97 μm) to the final stabilized state (PV: 2.76 μm), demonstrating its capability to provide rapid and accurate planar process feedback. This paper also uses a coordinate measuring machine to measure the final surface shape of the guideway. However, the coordinate measuring machine has some limitations, which are not discussed in detail in this paper. For details, please refer to Appendix A.

## 5. Conclusions

This study addresses the critical challenge of high-precision flatness measurement for large-scale, low-reflectivity ceramic surfaces by proposing a novel integrated approach combining oblique-incidence laser interferometry with sub-aperture stitching technology. The key innovations and contributions are summarized as follows:

(1) Methodological Advancements:

The oblique-incidence configuration significantly enhances interference signal contrast on rough ceramic surfaces by optimizing the incident angle, overcoming the reflectivity limitations of conventional normal-incidence interferometry.

Sub-aperture stitching extends the effective measurement range beyond the physical aperture of the interferometer, enabling full-surface characterization of elongated components while maintaining micrometer-level resolution.

(2) Validation and Performance:

Numerical simulations demonstrated robust reconstruction accuracy for surfaces with flatness values spanning 1–20 µm, revealing systematic errors dominated by mirror figure inaccuracies.

Experimental validation on a 1050 mm × 130 mm SiC guideway achieved complete surface mapping with PV 2.76 μm and RMS 0.59 μm. The method exhibited high consistency with traditional techniques in polished regions with PV deviation of 0.33 μm and RMS deviation of 0.04 µm.

(3) Practical Significance:

The system enabled quick monitoring of a 39-h lapping process, capturing flatness convergence from an initial PV 13.97 μm to a final PV 2.76 μm. This capability provides critical in-process feedback for ultra-precision manufacturing, reducing reliance on post-hoc metrology and accelerating production cycles.

Unlike existing methods limited to polished surfaces, this technique accommodates heterogeneous roughness distributions, making it uniquely suited for guiding multi-stage machining workflows.

(4) Industrial Applications and Outlook:

The method holds immediate promise for semiconductor manufacturing equipment, precision machine tools, and aerospace systems where large ceramic guideways demand sub-micrometer flatness control. Its adaptability to rough surfaces further supports quality assurance in the additive manufacturing of ceramic components.

Future work will focus on minimizing mirror-induced errors through mirror calibration or computational compensation and extending the framework to other large-scale surface metrology.

## Figures and Tables

**Figure A1:**
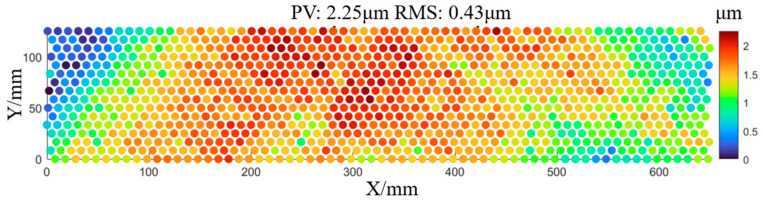
Coordinate measuring data chart.

## Data Availability

The raw data supporting the conclusions of this article will be made available by the authors on request.

## Appendix A

Compared with optical measurement methods, coordinate measuring machines (CMMs) have two significant limitations, as shown in Figure A1:

Disadvantages in sampling density and efficiency: The sampling density of CMMs is much lower than that of the stitching-oblique incidence interferometry and traditional plane laser interferometry. Within the measurement range of 130 mm × 650 mm, the data volume of CMMs (1040) is only a tiny part of that of the two optical methods (632,660 and 1,790,990). Moreover, the measurement efficiency is low, with a single measurement taking 1.5 h, which is much longer than the 5 min required for optical measurement.

Accuracy is restricted by sample size: For large-sized samples over 1 m, the measurement error of ordinary CMM exceeds 3 microns, and even high-precision models (such as those produced by Zeiss) have a maximum accuracy of only 0.3 microns.

Therefore, although the measurement data of CMMs are close to the results of the method proposed in this paper, this paper does not discuss them in detail due to the above-mentioned defects.
